# Crystal Structure and Computational Modeling of the Fab Fragment from a Protective Anti-Ricin Monoclonal Antibody

**DOI:** 10.1371/journal.pone.0052613

**Published:** 2012-12-19

**Authors:** Zhiyu Zhao, David Worthylake, Louis LeCour, Grace A. Maresh, Seth H. Pincus

**Affiliations:** 1 The Research Institute for Children, Children’s Hospital, New Orleans, Louisiana, United States of America; 2 The Louisiana Optical Network Initiative Institute, Baton Rouge, Louisiana, United States of America; 3 Department of Computer Sciences, The University of New Orleans, New Orleans, Louisiana, United States of America; 4 Department of Biochemistry and Molecular Biology, Louisiana State University Health Sciences Center, New Orleans, Louisiana, United States of America; 5 Departments of Pediatrics and Microbiology, Immunology, and Parasitology, Louisiana State University Health Sciences Center, New Orleans, Louisiana, United States of America; University of Michigan, United States of America

## Abstract

**Background:**

Many antibody crystal structures have been solved. Structural modeling programs have been developed that utilize this information to predict 3-D structures of an antibody based upon its sequence. Because of the problem of self-reference, the accuracy and utility of these predictions can only be tested when a new structure has not yet been deposited in the Protein Data Bank.

**Methods:**

We have solved the crystal structure of the Fab fragment of RAC18, a protective anti-ricin mAb, to 1.9 Å resolution. We have also modeled the Fv structure of RAC18 using publicly available Ab modeling tools Prediction of Immunoglobulin Structures (PIGS), RosettaAntibody, and Web Antibody Modeling (WAM). The model structures underwent energy minimization. We compared results to the crystal structure on the basis of root-mean-square deviation (RMSD), template modeling score (TM-score), Z-score, and MolProbity analysis.

**Findings:**

The crystal structure showed a pocket formed mainly by AA residues in each of the heavy chain complementarity determining regions (CDRs). There were differences between the crystal structure and structures predicted by the modeling tools, particularly in the CDRs. There were also differences among the predicted models, although the differences were small and within experimental error. No one modeling program was clearly superior to the others. In some cases, choosing structures based only on sequence homology to the crystallized Ab yielded RMSDs comparable to the models.

**Conclusions:**

Molecular modeling programs accurately predict the structure of most regions of antibody variable domains of RAC18. The hypervariable CDRs proved most difficult to model, particularly H chain CDR3. Because CDR3 is most often involved in contact with antigen, this defect must be considered when using models to identify potential contacts between antibody and antigen. Because this study represents only a single case, the results cannot be generalized. Rather they highlight the utility and limitations of modeling programs.

## Introduction

More than 250 mouse antibody (Ab) structures have been deposited in the RCSB Protein Data Bank (PDB; www.rcsb.org). This database, by allowing structural comparisons among Abs, has advanced the application of computational methods to predict their 3-D structures [1]–[5]. The amino acid (AA) sequences of an Ab’s heavy chain and light chain variable regions are provided to a modeling tool, resulting in the output of the probable 3-D coordinates. Two approaches are used in Ab modeling: homology modeling and ab initio (or de novo) modeling. Homology modeling uses 3-D structures of protein molecules with similar sequences as “templates” and produces a structure based on the template structures in conjunction with the AA differences between the template and the modeled sequence. Sequence alignment tools and sequence databases are often needed in homology modeling to discover the sequences to be used as templates, and structure databases are used to provide the coordinates of structures with closely related sequences. Sometimes, minor refinements such as those for side chains are applied to increase the prediction accuracy. As the overall fold of Abs is highly conserved, homology modeling performs quite well in accurately predicting the structure of the framework of the Fv region.

The complementarity determining regions (CDRs) of an Ab are necessarily variable in structure, and homology modeling is less successful here because of low sequence similarity in these regions and a corresponding structural divergence in the template. Therefore, the modeling of CDR loops is much more challenging and the resulting models of these regions are typically less accurate. There are two major loop-modeling methods currently used: loop grafting and *de novo* modeling. In essence, loop grafting directly copies the crystal structure coordinates of a known loop of similar length, although the sequence similarity may be quite low. This method works fairly well for CDR L1, L2, L3, H1 and H2 loops, but is less accurate in predicting the non-canonical structure of the H3 loop. Loop grafting, because it uses existing loop conformations as a starting point, has the potential to introduce structural bias into the final model. Another general method, *de novo* modeling, does not rely on existing structural templates for the loop regions. This method can therefore be utilized to predict, without bias, the conformation of these important Ab features. These approaches are utilized in publicly-available, web-based, Ab modeling tools, such as Web Antibody Modeling (WAM) [1], RosettaAntibody [5], and Prediction of Immunoglobulin Structures (PIGS) [4], and commercial software products such as Accelrys Discovery Studio [2] and Molecular Operating Environment (MOE) [3].

In this manuscript we compare results obtained using different Ab modeling tools with the actual X-ray crystallographic structure (1.9 Å) of the neutralizing anti-ricin mAb RAC18 [6]. Ricin toxin, derived from the castor bean *Ricinus communis*, is a prototypic A-B toxin. Its use as a bioterrorist weapon is of considerable concern. The crystal structure of ricin has been solved by X-ray crystallography at 2.5 Å [7] (PDB ID: 2AAI). In previous work [6], we made 43 mAbs to ricin toxin A-chain (RTA), B-chain and compound determinants on both chains. RAC18, directed against the A chain, has the greatest neutralization activity, *in vivo* protection, and highest binding avidity. Defining the structure of this Ab as it binds antigen (Ag) will make possible the design of higher affinity and better therapeutic Abs. The studies reported here represent initial steps toward that aim.

Because Ab modeling programs utilize PDB structures as the basis of homology fitting, and algorithms are refined as the data bank grows, it is not possible to test the predictions of modeling programs against structures already deposited in that database without the risk of self-reference. The only fair test is to compare the predictions to as yet unreported structures. In this report, we have used the RAC18 crystal structure in that way. Here we show differences among Ab modeling programs, and more importantly their deviation from the crystal structure. Structures were compared using a panel of different tools. The crystal structure and the models were also compared to the structures of those Abs with closest sequence homology found in the PDB.

## Materials and Methods

### RAC18 Ab and Fab

The murine RAC18 mAb, a highly protective and neutralizing anti-ricin A chain Ab, has been described elsewhere [6]. Hybridoma cells were grown in RPMI-1640 medium (Invitrogen, Grand Island, NY) supplemented with L-Glutamine, Gentamycin (Invitrogen), Penicillin-G, Oxalacetic acid, Pyruvic acid, Insulin (Sigma, St. Louis, MO) and 10% low IgG fetal bovine serum (FBS; Invitrogen). Abs were purified using protein G sepharose chromatography (Sigma) and elution by 0.2 M glycine-HCl pH 2.8. MAb was immediately neutralized with 2 M Tris base, and dialyzed against PBS. Nucleic acid sequences of the genes encoding the heavy and light chain variable regions of RAC18 have been deposited in GenBank (GenBank accession nos. **DQ164183.1** and **GQ165714.1**). Table 1 presents the features of the CDR regions of RAC18. The CDR loops and canonical structures were assigned using the Chothia criteria [8]–[11].

**Table 1:** Sequence Features of RAC18.

	Light Chain Variable Region (VL)	Heavy Chain Variable Region (VH)	
Total Length	109	118	
Framework Length	82	81	
V-Gene Family^a^	Vκ 19	VH J558	
J Gene	Jκ 5	JH 2	
Sequence of CDR1 (L1, H1)	_24_KASQDVTSAVA_34_	_26_GYTFTDYYVN_35_	
CDR1 Canonical Structure^b^	2	1	
Sequence of CDR 2 (L2, H2)	_50_SASYRYT_56_	_50_LIIPSNGGTTYNQKFRG_66_	
CDR2 Canonical Structure	1	2 or 3	
Sequence of CDR 3 (L3, H3)	_89_QQHYGTPLT_97_	_99_RGLTGALFAY_108_	
CDR L3 Canonical Structure	1		
Fv Length	227		

a Assignment by IgBlast http://www.ncbi.nlm.nih.gov/igblast/.

b Assigned according to references [10], [11], using the website http://www.bioinf.org.uk/abs/chothia.html.

Fab fragments of the RAC18 mAb were prepared by digestion with immobilized papain (Pierce, Rockford, IL). The reaction was carried out in sample buffer (20 mM sodium phosphate and 10 mM disodium EDTA, pH 7.0). The Ab was concentrated to 10 mg/ml in sample buffer. Papain beads were pre-activated by incubation at 37 °C for 30 minutes in digestion buffer (sample buffer +0.05 M L-Cysteine). Pre-activated beads were washed with sample buffer to remove cysteine and then 0.4 ml packed pre-activated papain beads were added per 10 mg of intact RAC18 Ab and incubated with shaking overnight at 37 °C. The reaction was terminated by the addition of 10 mM Tris-HCl, pH 8.0, to a final concentration of 5 mM. Fab fragments were separated from intact Ab and Fc fragments by Protein A (Sigma) chromatography. The purified Fab fragments were dialyzed against crystallization buffer (25 mM NaCl, 10 mM Tris-HCl and 1 mM EDTA, pH 7.4) and concentrated to 10 mg/ml with a 10 kDa molecular weight cutoff centrifugal filter (Amicon, Millipore, Ireland). Purified Fab fragments, in both reduced and non-reduced forms, were characterized by microcapillary electrophoresis (Agilent, GE Healthcare, Piscataway, NJ). The non-reduced result showed a band of molecular weight 47 kDa while the reduced showed bands of 28 kDa and 25 kDa. Fab fragments were further purified by Superose 12 (GE Healthcare) size fractionation FPLC.

### Fab Crystallization

Initial crystallization screens were performed with PEGs, PEGs II, and Ammonium Sulfate screening sets (Qiagen, Valencia, CA). The screens were performed with vapor diffusion in sitting drops at both 4 °C and room temperature. Crystals were first obtained in condition #63 of the PEGs Suite (0.2 M lithium nitrate and 20% w/v PEG 3350) at 4 °C. Crystals appeared after 10 days. Optimization of this condition resulted in a final well solution of 95–98 mM lithium nitrate, 20% w/v PEG 3350. A 2∶1 ratio of well solution to RAC18 protein yielded large, high quality crystals in approximately 2 weeks. For cryoprotection prior to data collection at 100 K, drops containing promising crystals were adjusted to mother liquor plus 10% (v/v) glycerol. The well solution was then adjusted to mother liquor plus 20% v/v glycerol and allowed to equilibrate by vapor diffusion with the drop overnight. The following day, a cryoprotected crystal was lassoed in a rayon loop (Hampton Research, Aliso Viejo, CA) and flash-cooled in liquid N_2_, prior to data collection. A Microstar generator equipped with Helios focusing optics was used as the X-ray source and data were collected using a Platinum 135 CCD camera (Bruker AXS). Data were integrated and scaled with Proteum2 software (Bruker AXS). RAC18 crystals belong to the orthorhombic spacegroup P2_1_2_1_2_1_ with unit cell dimensions a = 39.6 Å, b = 85.9 Å, and c = 130.1 Å. Volume considerations indicated that one 47 kDa Fab fragment occupied the crystal asymmetric unit (47.9% solvent).

### Crystal Data Collection, Structure Solution and Refinement

The structure of the RAC18 Fab was determined by molecular replacement using the program MOLREP [12] of the CCP4 suite [13]. The atomic coordinates of the heavy and light chains from the Fab fragments of mAbs BION-1 (PDB ID: 1EGJ chain H) and anti- SRPγ (PDB ID: 3DIF chain A), respectively, were utilized in this exercise. The globular domains of these models (residues 1–107 and 110–213 of 3DIF_A; residues 1–118 and 122–220 of 1EGJ_H), including all side chains, were used as the search models. Solutions for all domains of the Fab molecule in the asymmetric unit were easily determined. At this point, CNS [14] refinement was used to perform rigid body refinement, followed by simulated annealing with torsion angle dynamics. Model building was completed with alternating cycles of CNS automated positional and individual temperature factor refinement interspersed with manual model adjustments made using the interactive graphics program O [15]. The geometry of the final model was analyzed with PROCHECK [16]. The final model consists of 434 residues and 382 waters (Table 2). The model has good geometry with 90.7% of non-glycine residues in the most favored region and 9.0% in additional allowed regions of the Ramachandran plot. One residue, alanine 51 of L2, is occupying a position just outside of the generously allowed region of the Ramachandran plot. However, since backbone and side chain atoms of this residue are quite well represented in the composite omit 2Fo-Fc map and the temperature factors of atoms in this residue are much lower than the mean for protein atoms in this structure, there was no attempt made to manually adjust the backbone torsion angles. The atomic coordinates can be accessed at PDB (ID4H20).

**Table 2:** Data Collection and Refinement Statistics.

Data Collection	
Resolution (Å)	14.80–1.9
Measured Reflections (#)	154,753
Unique Reflections (#)	34,728
Data Redundancy	4.2 (1.7)^a^
Data Completeness (%)	96.6 (87.0)
Rint (%)^b^	4.28 (9.34)
I/sigI	22.67(7.68)
**Refinement**	
R factor^c^/R free^d^	19.5/22.3
Free R test set size (#/%)	2,070/5.8
Number of protein atoms	3,307
Number of solvent atoms	382
Rmsd bond lengths (Å)	0.005
Rmsd bond angles (°)	1.7
Mean B factor (Å^2^) protein/solvent	13.97/22.47
Rmsd B factors (Å^2^) bonded (main chain/side chain)	1.27/2.21

a Data in parenthesis pertain to the highest resolution shell (2.0 Å-1.9 Å).

b Rint = ∑|I - <I>|/∑I, where I is the observed intensity of a measured reflection and <I> is the mean intensity for all observation of symmetry-related reflections.

c R factor = Σ |F*_oh_* – F*_ch_*|/Σ F*_oh_*, where F*_oh_* and F*_ch_* are the observed and calculated structure factor amplitudes for the 32,658 reflections *h* that were used in structure refinement.

d R free = Σ |F*_oh_* – F*_ch_*|/Σ F*_oh_*, where F*_oh_* and F*_ch_* are the observed and calculated structure factor amplitudes pertaining to the 2,070 reflections *h* that were not used in structure refinement.

### Ab Modeling Tools

We have compared the basic characteristics of the following Ab modeling tools: Discovery Studio, MOE, PIGS (website: http://www.biocomputing.it/pigs/), RosettaAntibody (website: http://antibody.graylab.jhu.edu/), and WAM (website: http://antibody.bath.ac.uk/). These are summarized in Table 3. We have chosen the latter three for our computational analyses because they are web-based and are free for academic users. The basic inputs of all three Ab modeling tools are the AA sequences of the VL and VH regions of an antibody. Besides the sequences, PIGS allows the user to input a list of PDB templates that should be excluded in the modeling. It also allows the user to select a VL or VH template from a list of PDB candidates, choose a loop grafting method, and apply a side chain modeling method. In our PIGS modeling of the RAC18 mAb we used default options. For RosettaAntibody, the user can choose whether or not to *de novo* model the CDR H3 loop, and whether or not to optimize the relative orientation of the light and heavy chains. In our modeling, we chose both H3 modeling and the orientation optimization. Also, RosettaAntibody outputted 10 ranked models and we used the top model in our comparison. For WAM, one can choose to manually align the input sequences with the known Ab sequences or to have the alignment done automatically. We chose automatic alignment. WAM allows the user to select a side chain building method and a final screening method. In addition, it has options especially designed for the CDR H3 loop. We used the website’s recommendations, including dead-end elimination for side chain modeling, the RMSD screen for final screening, use of the database search for non-canonical, non-H3 loops only, and use of tentative sequence-structure rules for H3 loops during searching when modeling our antibody. The structures predicted by the modeling tools were then subjected to energy minimization using knowledge based potentials [17] incorporated at the website: http://www.yasara.org/minimizationserver.htm.

**Table 3:** Features of Ab Modeling Tools.

	Discovery Studio	MOE	PIGS	RosettaAntibody	WAM
Modeling Methods	Homology modeling, loop grafting, side chain refinement and *de novo* loop modeling.	Homology modeling,loop grafting and sidechain refinement.	Homology modeling,loop grafting and sidechain refinement.	Homology modeling, loopgrafting and *de novo*loop modeling.	Homology modeling, loop grafting and side chain refinement.
Running Time	Not tested	Not tested	Minutes	Online version: Hours(Depending on the numberof jobs in the submissionqueue); Standalone version:Not tested.	Minutes but needs apassword to be sent fromthe ownerbefore downloading.
Web Server	No	No	Yes	Yes	Yes
Stand-alone Program	Yes	Yes	No	Yes	No
Software Suite	Yes	Yes	No	Yes	No
Free	No	No	Yes	Web server is free. Standalone program is included in the Rosetta suite and free for academic users only.	Free for academicusers only.

### Graphic Display, Comparisons and Calculations of 3-D Structures

All structures in this manuscript were displayed in PyMOL, version 1.4.1 [18]. PyMOL was also used to align structures, calculate the RMSD distance between them, and estimate the size of the Ag binding pocket in the RAC18 Fab. The RMSDs shown, except where noted, are the result of all-AA-C_α_ alignments using PyMOL’s “pair-fit” function, which are possible because each region has the same number of residues. Alignments were performed separately for each region indicated, rather than aligning the entire V-region and measuring the RMSD in the indicated region. However, when this is not possible because of length differences, we used PyMOL’s “super” function. The TM-score [19], Z score [20], [21] and MolProbity metrics [22], [23] were all calculated using their corresponding websites (http://zhanglab.ccmb.med.umich.edu/TM-score/, https://prosa.services.came.sbg.ac.at/prosa.php, and http://molprobity.biochem.duke.edu/). Both the RMSD and TM-score consider Cα backbone distances only. Z score is used to compare the energy of structures of the same size. Molprobity analysis evaluates the quality of structures by incorporating side-chain information. The modeling programs were compared statistically by calculating significance of differences between models and/or differences from the crystal structure using a two-tailed student's t test, paired for each of the CDRs and the framework regions.

## Results

### Crystallization of RAC18 Fab

**Figure 1:**
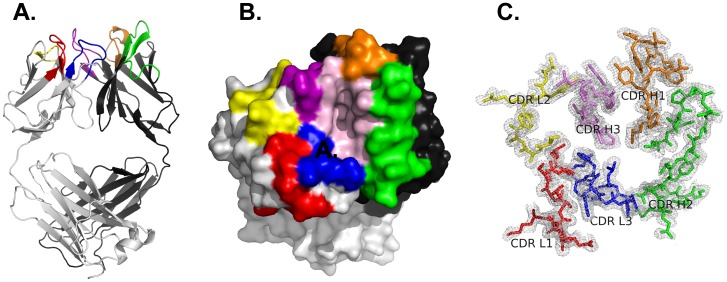
Three-dimensional crystal structure of the RAC18 Fab. A. Ribbon structure of the Fab. B. Surface diagram of a potential Ag-binding pocket. C. Electron density map of the CDR loops contoured at 0.5 σ. Color code: light gray (light chain), dark gray (heavy chain), red (CDR L1), yellow (CDR L2), blue (CDR L3), orange (CDR H1), green (CDR H2), purple (CDR H3). Residues lining the pocket are in pink; L chain 96L; H chain 32Y, 33Y, 35N, 47W, 50L, 97A, 98R, 99R, 105L, and 106F. Pocket size: height ∼8 Å, major axis ∼12 Å, minor axis ∼5 Å. Lining the pocket are 6 hydrophobic, and 5 hydrophilic (2 charged) residues.

The final refinement statistics of the RAC18 Fab crystal structure are listed in Table 2. The structure has been deposited in the PDB and has been assigned the RCSB ID code rcsb074918 and PDB ID code 4H20. Figure 1 shows the X-ray structure of the RAC18 Fab and highlights a pocket formed by 11 AA residues in or adjacent to its CDR regions. Among the 11 AAs, L96 is in CDR L3; the other 10 are part of the heavy chain. Heavy chain residues are Y32, Y33, and N35 in CDR H1; L50 in CDR H2, R99, L105; and F106 in CDR H3, and W47, A97, and R98 in the heavy chain framework. The shape of the pocket can be approximated as a half-ellipsoid with an estimated height of 8 Å, and major and minor axes of approximately 12 Å and 5 Å, respectively. There are 6 hydrophobic and 5 hydrophilic AAs in the pocket, and 2 hydrophilic AAs (R98 and R99) are charged. Although we have not identified the Ab combining site experimentally, it is a reasonable assumption that the pocket we describe, lined by residues from the CDRs, is likely to participate in binding to antigen. The composite omit 2Fo-Fc electron density map of the CDR regions of the RAC18 Fab is also presented in Figure 1.

### RMSD Differences Among Ab Modeling Tools and Crystal Structure

We have modeled the Fv region of the RAC18 mAb with the online Ab modeling tools PIGS, RosettaAntibody and WAM. The Yasara force field was then used to perform energy minimizations of these structures. We evaluated the performance of the modeling tools by measuring the RMSD between the *in silico* models and the 1.9 Å crystal structure of RAC18 we obtained experimentally. PyMOL was used to align the C_α_ backbone of each predicted model with that of the crystal structure and provide RMSD values. Table 4 compares the three modeling tools’ performances in various regions of RAC18. RosettaAntibody’s output PDB file includes only the first 106 AA residues in the VL region. In order to compare it with other modeling tools, we removed the last three residues from the VL of PIGS and WAM models, so that all the models are of the same size. The results indicate that PIGS and RosettaAntibody frequently have smaller RMSDs than WAM, but none of the differences between the models and crystal structures are statistically significant. Energy minimization of the model structures generally resulted in decreased overall RMSDs, but in some regions increases were observed. The predictions of WAM were most improved by energy minimization. The greatest RMSDs between the models and crystal were observed for the non-canonical CDR3. In this region, the de novo modeling of RosettaAntibody outperformed the loop grafting used by the PIGS and WAM.

**Table 4:** RMSD Distances Between Models and Crystal Structure.

	PIGS		RosettaAntibody		WAM		Smallest RMSD^b^	
Region	Before^a^	After^a^	Before	After	Before	After	Before	After
CDR L1	0.25^c^	0.14	0.21	0.19	0.26	0.37	Rosetta	PIGS
CDR L2	0.17	0.15	0.24	0.26	0.19	0.19	PIGS	PIGS
CDR L3	0.47	0.38	0.77	0.65	0.56	0.50	PIGS	PIGS
CDR H1	0.26	0.34	0.37	0.32	0.18	0.26	WAM	WAM
CDR H2	0.33	0.44	0.98	0.91	0.35	0.33	PIGS	WAM
CDR H3	2.15	2.16	1.45	1.42	1.88	1.83	Rosetta	Rosetta
VL	0.44	0.53	0.57	0.49	0.62	0.56	PIGS	Rosetta
VH	1.03	0.93	1.08	1.13	1.25	1.10	PIGS	PIGS
CDR L1-3	0.39	0.39	0.78	0.59	0.59	0.47	PIGS	PIGS
CDR H1-3	1.38	1.46	1.40	1.33	1.72	1.58	PIGS	Rosetta
L Chain Framework	0.44	0.55	0.37	0.41	0.61	0.57	Rosetta	Rosetta
H Chain Framework	0.79	0.47	0.79	0.92	0.80	0.63	PIGS	PIGS
Framework (L & H)	0.77	0.70	0.85	0.92	1.10	1.00	PIGS	PIGS
Fv	0.94	0.91	1.06	1.04	1.39	1.30	PIGS	PIGS

a Before or after energy minimization of the model structures.

b None of the comparisons among the models are significantly different from the others.

c Angstroms.

### Visualization of Backbone Alignments

**Figure 2:**
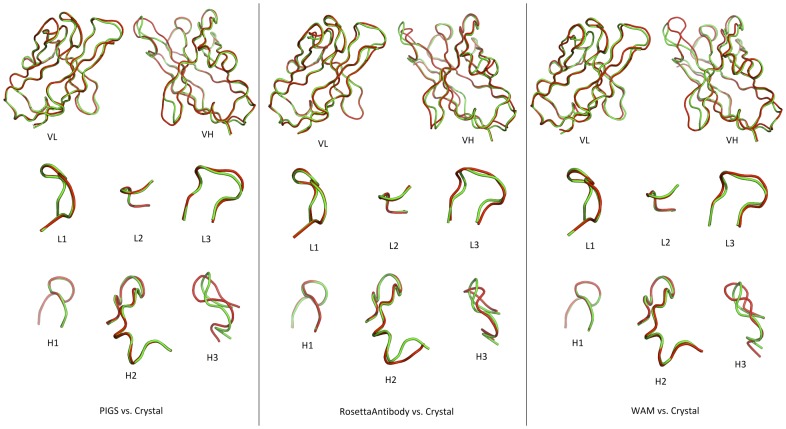
Pairwise Alignments between Models and the Crystal Structure of RAC18 Fab. Color code: The crystal is green and the models are red. Top: F_V_ domains. Bottom: CDRs of H and L chains. The models shown here are prior to energy minimization.

Figure 2 displays the C_α_ backbone alignments between predicted models and the crystal structure of the Fv region of the RAC18 Fab. Although there is a great deal of similarity, this figure shows that dissimilarities in a small region of the CDR H3 loop account for much of the differences in the RMSD values calculated for the crystal vs. PIGS or WAM. However this is often a critical region making contact with antigen, and correct CDR H3 predictions are necessary for efficacious use of modeling predictions.

### TM-score of Modeling Tools

Because RMSD is a raw, distance-based score that reflects the overall Cα-Cα displacement between the aligned structures, here we also report the TM-score (website: http://zhanglab.ccmb.med.umich.edu/TM-score/) for those alignments. The TM-score is a commonly used protein structure quality readout [19]. It is often used to evaluate the similarity between a predicted model and a native structure. TM-score is normalized to a value between 0 and 1, and it rewards a long contiguous alignment with low RMSD even if the overall alignment of the two structures displays regions of high RMSD variation. The TM-score calculation website allows for comparisons of structures of continuous residues in a single chain. So we calculated scores for L1, L2, L3, H1, H2, H3, VL and VH regions individually. The scores are shown in Table 5. Tools with the highest TM-score in the 8 tested regions are exactly those with the lowest RMSD; the two geometric scores verify each other quite well in the modeling of the RAC18 mAb.

**Table 5:** TM-Score of Comparison of Model to Crystal Structure.

		PIGS			RosettaAb			WAM			Highest^a^	
Region		Before^b^	After^b^		Before	After		Before	After		Before	After
CDR L1		0.81	0.93		0.86	0.88		0.81	0.7		Rosetta	PIGS
CDR L2		0.90	0.92		0.82	0.80		0.88	0.88		PIGS	PIGS
CDR L3		0.66	0.78		0.46	0.57		0.5	0.61		PIGS	PIGS
CDR H1		0.80	0.71		0.67	0.75		0.9	0.81		WAM	WAM
CDR H2		0.73	0.63		0.60	0.60		0.71	0.74		PIGS	WAM
CDR H3		0.29	0.28		0.44	0.50		0.27	0.23		Rosetta	Rosetta
VL		0.99	0.98		0.98	0.98		0.97	0.98		PIGS	N/A
VH		0.96	0.97		0.95	0.95		0.95	0.96		PIGS	PIGS

a None of the comparisons among the models are significantly different from the others.

b Before or after energy minimization of the models.

### Z-score of Modeling Tools

Both the RMSD and TM-score take advantage of the structures’ geometric information only. In order to take into account the structures’ apparent folded energy, we used Protein Structure Analysis-web (ProSA-web, website: https://prosa.services.came.sbg.ac.at/prosa.php) [20], [21] to calculate a Z-score for all the predicted models as well as the crystal structure of the RAC18. Here the Z-score of a protein structure is calculated as the difference between a protein’s energy and the average energy of structures of the same size in the PDB, divided by the energy standard deviation of those structures. Both the average energy and the standard deviation are constants; therefore, a Z-score is a representation of the energy of a structure with the idea being that a high Z-score (high energy) would be inconsistent with a thermodynamically relevant structure. As ProSA-web can output a plot showing the position of a particular Z-score among the Z-scores of other structures in the PDB, the Z-score also reflects the probable “correctness” of a protein structure. Because the Z-score is a measure of the folded energy of the structure, we only determined Z-scores of the non-minimized structures. In our calculations we found that all the Z-scores in Table 6 are within the reasonable range of Z-scores of structures in the PDB, as reported by ProSA-web. It is interesting to see that regions in the crystal structure do not always have the lowest energy as calculated by the Z-score. Actually among the 8 regions in the crystal structure, only 2 of them have the lowest Z-score, when compared to the models. It should be mentioned that structures with similar energies may sometimes achieve this by adopting different backbone conformations. For example, in Figure 2, Table 4, and Table 6, the CDR H2 of RosettaAntibody (blue) and that of WAM (yellow) both have Z-scores of 0.40 but the RMSD between RosettaAntibody and the crystal structure’s CDR H2 is 0.98 Å, while the RMSD between WAM and crystal structure is only 0.35 Å. Structures with quite different energies may look highly similar in their backbone conformations, eg. CDR L1 of PIGS, RosettaAntibody, and WAM all match that of the crystal structure very well, and their RMSDs are only 0.25, 0.21 and 0.26, respectively; however, their Z-scores are 0.86, 0.87, and 1.37, respectively, while the Z-score of the crystal structure is only 0.40.

**Table 6:** Z-Score of Modeling Tools.

	PIGS	RosettaAntibody	WAM	Lowest	Crystal(for reference)
L1	0.86	0.87	1.37	PIGS	0.40
L2	−0.98	−0.97	−1.12	WAM	−0.97
L3	−0.14	−0.45	−0.24	RosettaAntibody	−0.20
H1	−0.24	−0.46	−0.15	RosettaAntibody	−0.24
H2	0.26	0.40	0.40	PIGS	0.26
H3	1.09	0.34	2.07	RosettaAntibody	1.22
VL	−5.31	−5.04	−4.79	PIGS	−5.15
VH	−6.33	−6.28	−6.03	PIGS	−6.10

### MolProbity Analysis

Because both the RMSD and TM-score are calculated on the basis of Cα backbones and do not take side-chain conformations into account, we have performed a MolProbity analysis [22], [23] (website: http://molprobity.biochem.duke.edu/) on all the models (before and after energy minimization) as well as the crystal structure. The MolProbity profile evaluates the quality of experimental structures or predicted protein models with metrics including clash score, poor rotamers, Ramachandran outliers, Ramachandran favored, Cβ deviations >0.25 Å, MolProbity score, residues with bad bonds, and residues with bad angles. The MolProbity analysis has been applied to evaluate protein models generated at the 8^th^ Critical Assessment of Protein Structure Prediction experiment (CASP 8) [24], and to Ab structures by Almagro et al [25]. Table 7 reports the MolProbity results of the PIGS, RosettaAntibody, and WAM models. The MolProbity analysis of the crystal structure is shown in Table 7 for reference. We have used the MolProbity website’s default settings for all the structures analyzed. When necessary, hydrogen atoms were added to structures with the website’s recommended method before performing all-atom contact analysis. Of the metrics shown in Table 7, RosettaAntibody was most often best. Not unexpectedly, energy minimization of the models improved most parameters of the MolProbity analysis, in some cases quite markedly.

**Table 7:** Molprobity Analysis.

	PIGS		RosettaAb		WAM		Best		RAC 18 Crystal (for reference)
	Before	After	Before	After	Before	After	Before	After	
Clash Score (%)^a^	42.34 (7%)	0.59 (99%)	8.82 (78%)	0.59 (99%)	39.36 (8%)	0.59 (99%)	Rosetta	N/A	14.54 (54%)
Poor Rotamers^b^	9.04%	1.06%	0.53%	1.60%	12.22%	2.66%	Rosetta	PIGS	1.10%
Ramachandran Outliers^c^	1.82%	0.45%	0.45%	0.00%	0.91%	0.45%	Rosetta	Rosetta	0.91%
Ramachandran Favored^d^	91.36%	96.36%	92.73%	98.64%	92.27%	95.00%	Rosetta	Rosetta	94.55%
Cβ Deviations >0.25 Å^e^	0	0	0	0	8	0	Rosetta, PIGS	N/A	0
Residues with bad bonds^f^	0.00%	0.00%	2.23%	0.00%	0.45%	0.00%	PIGS	N/A	0.00%
Residues withBad Angles^g^	1.34%	0.45%	2.68%	0.00%	0.89%	0.89%	WAM	Rosetta	0.45%
MolProbity Score^h^ (%^a^)	3.34 (12%)	0.96 (100%)	1.93 (79%)	0.85 (100%)	3.38 (11%)	1.37 (98%)	Rosetta	Rosetta	2.07 (62%)

a 100% (100^th^ percentile) is the best; 0% (0^th^ percentile) is the worst.

b Goal: <1%.

c Goal: <0.2%.

d Goal: >98%.

e Goal: 0.

f Goal: 0%.

g Goal: <0.1%.

h MolProbity score: the lower, the better; it is a composite index calculated as a combination of clash score, percentage of rotamer outliers, and percentage of Ramachandran favored regions.

### Comparison of RAC18 Crystal and Modeled Structures to Other Existing Structures in PDB

One potential explanation for differences among structures predicted by the modeling tools is that the models have chosen different templates upon which to build the models. In Table 8 we show that this is indeed the case, there was no overlap among the templates chosen by each tool. We also compared the RMSD distances observed between the template and the crystal, and between the (energy-minimized) model and the crystal. Surprisingly the template had smaller RMSDs than the model in 3 out of 8 regions for PIGS, 5 out of 8 for WAM, and 4 out of 8 for RosettaAntibody. Given this result, we next compared the the crystal structure of RAC18 to structures in PDB with the highest sequence homology to RAC18. We found that the RMSDs of Abs chosen solely on the basis of sequence homology, calculated over the entire V region, were not much different than those obtained from the modeling tools (Table 9 vs Table 4). We reiterate that because of differences in AA lengths, RMSD calculations were performed differently in these two tables. Given this caveat, these data do suggest that the use of modeling tools offers only modest improvements over simple, sequence-based comparisons.

**Table 8:** Templates Used to Create Models.

		Light Chain				Heavy Chain			
Modeling tool	Region	PDB code	Sequence identity^a^	RMSD template^b^	RMSD model^c^	PDB code	Sequence identity^a^	RMSD template^b^	RMSD model^c^
PIGS	CDR 1	2NR6:C	8/11	0.27	0.14	1F11:B	8/10	0.26	0.34
	CDR 2	2NR6:C	7/7	0.17	0.15	1F11:B	12/17	0.31	0.44
	CDR 3	2NR6:C	6/9	0.41	0.38	1BFO:B	5/10	2.19	2.16
	Framework	2NR6:C	79/82	0.86	0.55	1F11:B	75/81	0.40	0.47
WAM	CDR 1	1IAI:L	9/11	0.25	0.37	1EGJ:H	8/10	0.17	0.26
	CDR 2	1IAI:L	6/7	0.17	0.19	1EGJ:H	13/17	0.31	0.33
	CDR 3	1IAI:L	6/9	0.59	0.50	1EGJ:H	5/10	2.07	1.83
	Framework	1IAI:L	77/82	0.98	0.57	1EGJ:H	69/81	0.60	0.63
Rosetta	CDR 1	1H8N	9/11	2.65	0.19	1AD9	9/10	0.28	0.32
Antibody	CDR 2	1L7I	7/7	0.23	0.26	1KTR	12/17	0.93	0.91
	CDR 3	1P7K	8/9	0.80	0.65	1CLY^d^	7/10	1.39	1.42
	Framework	1I3G	72/79^e^	0.37	0.41	1I3G	67/81	0.95	0.92

a Number of identical AA residues in template and RAC18.

b RMSD difference between structure of template and structure of RAC 18.

c RMSD difference between energy-minimized model and structure of RAC 18.

d This template was not used in creating the model, rather de novo modeling was performed.

e This template has fewer AA residues than RAC18.

**Table 9:** Antibodies with Sequence Homology to RAC 18 in PDB.

L Chain (109 AAs)				H Chain (118 AAs)			
	Sequence Identity by IgBlast	RMSD a	# Aligned		Sequence Identity by IgBlast	RMSD	# Aligned
3IU4:L	95.8% (91/95)	0.54	104	1F11:B b	88.7% (86/97)	0.76	112
3DIF:A	94.7% (90/95)	0.42	104	1KTR:H	83.3% (80/96)	1.13	104
2NR6:C b	93.7% (89/95)	0.39	104	2PCP:B	81.6% (80/98)	1.20	112
1IAI:L c	91.6% (87/95)	0.60	104	3RHW:F	83.7% (82/98)	1.44	112
3GK8:L	90.5% (86/95)	0.51	104	1EGJ:H c	83.7% (82/98)	1.10	112
1ZTX:L	88.4% (84/95)	0.60	104	1A6T:B	81.6% (80/98)	0.76	112
3S88:L	86.3% (82/95)	0.66	104	2G2R:H	82.3% (79/96)	1.37	112
3IY0:L	83.2% (79/95)	0.53	104	1F3D:H	80.4% (78/97)	1.78	112
1I3G:L	83.2% (79/95)	0.41	104	1XIW:D	81.6% (80/98)	1.90	112
1NCA:L	83.2% (79/95)	0.83	104	4F33:B	82.5% (80/97)	1.26	112

a RMSDs between crystal structure of homologous structures and RAC 18.

RMSDs calculated over entire VL or VH using Pymol “super” function.

b Used by PIGS as the templates for modeling.

c Used by WAM as the templates for modeling.

## Discussion

The Ab crystallization and modeling work described here was performed as an initial step towards producing higher affinity Abs through designed alterations of Ab structure. Ab modeling tools of high accuracy clearly would aid this process immensely. Therefore, we performed a systematic comparison of the structural predictions of three publicly available on-line tools with the crystal structure of the RAC18 Ab. To avoid self-reference, such analyses can only be performed on unreported structures, because the programs use PDB structures to create homology models. We have found that: (1) All three modeling tools utilized different templates and returned somewhat different structures. PIGS has the best performance in predicting the 3D structure of the RAC18 Fab in terms of RMSD and TM-Score, and its overall performance in predicting the VL and VH regions measured by the Z-score is also the best, although differences are within experimental error and probably not significant. (2) Energy minimization of the model structures does not significantly improve RMSD or TM scores. (3) The templates used by the modeling tools and Abs with high sequence homology to RAC18 had RMSD differences on the same order of those of the structures returned by the modeling tools. (4) RosettaAntibody is the best at energy-minimization based loop modeling, especially the complicated CDR H3 modeling, because the H3 structure from RosettaAntibody has the smallest RMSD, highest TM-score, and lowest Z-score. (5) Z-score by itself seems to not be a very reliable means to predict the veracity of modeled structures, since the crystal structure does not have the lowest Z-score. (6) MolProbity analysis reveals the weakness of PIGS and the strength of RosettaAntibody in performing side-chain refinement. However these differences disappear when MolProbity analyses are performed on energy minimized structures.

The native structure is not necessarily the one with the lowest energy among potential models. This implies that evaluation of Ab modeling tools based upon model energies may be misleading, and that the details of the energy-minimizing algorithm may lead to errors in the predicted structure in some cases. In addition, structures with similar energies may have noticeably different conformations and structures with different energies may have very similar conformations (see Figure 2, Table 4, and Table 6), thus adding to the difficulty in assessing the accuracy of energy based Ab modeling methods.

Almagro et al [25] have performed a similar set of analyses, comparing unpublished Ab crystal structures to results obtained with molecular modeling programs. They compared tools including Discovery Studio, MOE, PIGS, and RosettaAntibody over a set of 9 Ab structures (4 mouse, 1 rat, 3 human and 1 humanized) whose crystal structures were solved by X-ray crystallography at a resolution range 1.5 Å –2.3 Å. Best models outputted by those tools were assessed with two criteria: MolProbity and RMSD. No TM-score or Z score values were calculated for those benchmark Abs. Three MolProbity metrics (clash score, Ramachandran favored, and MolProbity score) were reported for each model as well as the crystal structure of each benchmark Ab. Similar to our RMSD analysis, they calculated RMSD values between models and their crystal structures for regions such as entire Fv, frameworks, VL, VH, six CDR loops, etc. Their results also showed that PIGS has the best overall RMSD performance in antibody modeling, and RosettaAntibody is better in side-chain refinement as measured by MolProbity analysis. In addition, according to Almagro et al 25, the average RMSD values of the non-canonical H3 loop were the largest among all the tested regions of the benchmark Abs. This agrees with our observation about RAC18. The similarity of their results and ours on different Abs suggests that the conclusions drawn about the relative merits of Ab modeling programs could be more broadly applicable than just the observations of our single case study. However, among their 8 benchmark Abs where H3 RMSD values are present for both PIGS and RosettaAntibody, PIGS values range from 2.2 to 4.2 Å with an average of 3.2±0.7 Å. RosettaAntibody values range from 1.8 to 5.5 Å with an average of 3.3±1.3 Å. Therefore, RosettaAntibody did not outperform PIGS in modeling the H3 of those benchmark Abs, as opposed to our observations of H3 of RAC18.

We have solved the 1.9 Å crystal structure of the Fab fragment of RAC18, a therapeutically important Ab with protective efficacy against the plant toxin ricin, an agent of bioterrorism concern. We have identified a potential Ag-binding pocket, whose shape can be simulated by a half-ellipsoid. The pocket is approximately half hydrophobic, half hydrophilic, and contains two charged residues. We have previously mapped the epitope in RTA that is bound by RAC18, using random peptide phage display libraries. We identified QXXWXXA as the principal motif. There is only one tryptophan in RTA (W211) and this tryptophan is part of the RTA enzyme active site. RAC18’s binding of this epitope is consistent with its ability to inhibit the enzymatic function of RTA *in vitro* [6]. However, because residue W211 is at the bottom of a cleft in RTA, an RTA-RAC18 interaction involving W211 is difficult to visualize. One way for RAC18 to make contact with W211 is via exposed Ab residues that project sufficiently outward when Ag is bound. Glutamine is also part of the phage display epitope motif, and residue Q98 does protrude from the surface of RTA, making it available for Ab recognition. The pocket structure made by the RAC18 CDRs contains basic residues (R98 and R99) and is partially hydrophilic, and might support positive Q98 interactions. However, it must be emphasized that these are only suppositions based on epitope mapping studies and our assumption that the pocket lined by CDR residues present in our crystal structure plays a role in antigen-binding. Because our goal is to modify the structure of RAC18 to increase its affinity of binding to RTA, future studies will include resolution of the crystal structure of the RAC18-RTA complex, and analysis of Ab-Ag docking programs as we have done here. As Ab modeling and Ab-Ag docking tools improve, we will attempt to predict AA mutations that could produce higher affinity anti-ricin mAbs based on RAC18.
